# Tumour Suppressive Function and Modulation of Programmed Cell Death 4 (PDCD4) in Ovarian Cancer

**DOI:** 10.1371/journal.pone.0030311

**Published:** 2012-01-17

**Authors:** Na Wei, Stephanie S. Liu, Karen K. L. Chan, Hextan Y. S. Ngan

**Affiliations:** Department of Obstetrics & Gynaecology, Queen Mary Hospital, the University of Hong Kong, Hong Kong, Hong Kong; University of Birmingham, United Kingdom

## Abstract

**Background:**

Programmed cell death 4 (PDCD4), originally identified as the neoplastic transformation inhibitor, was attenuated in various cancer types. Our previous study demonstrated a continuous down-regulation of PDCD4 expression in the sequence of normal-borderline-malignant ovarian tissue samples and a significant correlation of PDCD4 expression with disease-free survival. The objective of the current study was to further investigate the function and modulation of PDCD4 in ovarian cancer cells.

**Principal Findings:**

We demonstrated that ectopic PDCD4 expression significantly inhibited cell proliferation by inducing cell cycle arrest at G_1_ stage and up-regulation of cell cycle inhibitors of p27 and p21. Cell migration and invasion were also inhibited by PDCD4. PDCD4 over-expressing cells exhibited elevated phosphatase and tensin homolog (PTEN) and inhibited protein kinase B (p-Akt). In addition, the expression of PDCD4 was up-regulated and it was exported to the cytoplasm upon serum withdrawal treatment, but it was rapidly depleted via proteasomal degradation upon serum re-administration. Treatment of a phosphoinositide 3-kinase (PI3K) inhibitor prevented the degradation of PDCD4, indicating the involvement of PI3K-Akt pathway in the modulation of PDCD4.

**Conclusion:**

PDCD4 may play a critical function in arresting cell cycle progression at key checkpoint, thus inhibiting cell proliferation, as well as suppressing tumour metastasis. The PI3K-Akt pathway was implied to be involved in the regulation of PDCD4 degradation in ovarian cancer cells. In response to the stress condition, endogenous PDCD4 was able to shuttle between cell compartments to perform its diverted functions.

## Introduction

PDCD4 was originally identified as the neoplasmic transformation inhibitor in the JB6 mouse epidermal cell line model [1]. PDCD4 transgenic mice showed lower tumour incidence and papilloma-to-carcinoma conversion frequency [2]. Later reports have implied PDCD4's inhibitory role on protein translation through inhibition of eukaryotic initiation factor 4A (eIF4A) helicase, as well as interfering with the association of eIF4A with eIF4G, resulting in the failure of formation of translation initiation complex [3], [4], [5]. Since then, several studies have been conducted to investigate the role of PDCD4 during tumourigenesis. PDCD4 was found to be capable of regulating transcription. Over-expression of PDCD4 resulted in suppressed carcinoid cell proliferation through repressing the transcription of the mitosis-promoting factor cyclin-dependent kinase (CDK)1/cdc2 via up regulation of p21^Waf1/Cip1^ [6], [7]. PDCD4 inhibited colon cancer cell invasion through suppressing mitogen-activated protein kinase kinase kinase kinase 1 (MAP4K1), leading to suppressed AP-1 dependent transcription [8]. The role of PDCD4 in cell apoptosis has also been investigated in different studies. PDCD4 was suggested to be a proapoptotic molecule involved in transforming growth factor beta-1 (TGF beta-1) induced apoptosis in hepatocellular carcinoma (HCC) [9]. Diminished PDCD4 expression deregulated the normal DNA-damage response, thus preventing DNA-damaged cells from undergoing apoptosis [10].

Despite the tumor suppressor properties mentioned above, the role of PDCD4 in tumor progression has been suggested to be cell type specific [11]. Over-expression of PDCD4 had no effect on either proliferation or apoptosis in HEK293 cells [12], as well as in RKO colon cancer cells [8]. Previous studies reported the depleted PDCD4 expression in cancer compared with normal tissues [13], [14], [15], and PDCD4 was targeted for degradation during tumour promotion [16], however, the mechanisms for the modulation of PDCD4 was not clear yet.

The investigations on the role of PDCD4 in ovarian cancer carcinogenesis were rather limited. According to our previous findings, loss of PDCD4 expression was found in the borderline and malignant ovarian tissue samples, and associated with an adverse disease outcome [17]. To further investigate the role of PDCD4 in ovarian cancer, in the current study, we examined the potential tumour suppressor functions of PDCD4 in ovarian cancer cells, and the plausible mechanism that regulates PDCD4.

## Results

### PDCD4 inhibited ovarian cancer cell proliferation and cell cycle progression

**Figure 1:**
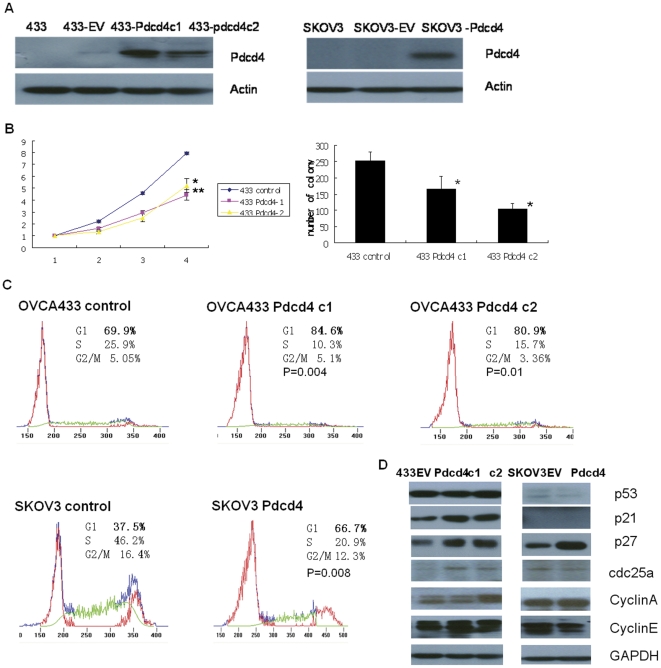
PDCD4 inhibited cell proliferation and cell cycle progression through up regulation of p21 and p27. (A) PDCD4 over-expressing stable clones 433-PDCD4c1, 433-pdcd4c2, and SKOV3-PDCD4, were established in ovarian cancer cells OVCA433 and SKOV3. 433-EV and SKOV3-EV: GFP over-expressing empty vector stable clones for control. (B) 433 PDCD4c1 and 433 PDCD4c2 exhibited significant slower proliferation rate compared with control (*p<0.01 and **p<0.05, respectively) according to XTT (left panel) and clonogenic assay (p<0.05) (right panel). (C) PDCD4 induced cell cycle arrest at G1 stage. Representative data are from one of the three independent experiments. The percentages of cells that were in G1 stage for OVCA433 c1 and c2 were 84.6% (±2.1%) and 80.9% (±2.2%), respectively. Both were significantly higher compared with control (69.9%±3.1%) (p = 0.004 and P = 0.01, respectively). The percentage of cells that was in G1 stage for SKOV3-PDCD4 was 66.7% (±1.8%), which was significantly higher compared with control (37.5%±2%) (p = 0.008). (D) PDCD4 over-expressing stable clones as well as control empty vector stable clones were maintained in MEM with 10% FBS, and then harvested for protein extraction. Protein expressions of a panel of cell cycle regulators including p53, p21, p27, cdc25a, cyclinA and cyclinE were analyzed. The intensity of the band was determined by densitometric scanning. The quantification of the bands was presented in Data S1. GAPDH was included as internal loading control. Three independent experiments were performed.

To investigate the function of PDCD4 in ovarian cancer, two PDCD4 over-expressing stable clones 433-PDCD4c1 and 433-PDCD4c2, were established in ovarian cancer cell OVCA433. One PDCD4 over-expressing stable clone SKOV3-PDCD4, was established in ovarian cancer cell SKOV3 (Figure 1A). The establishment of PDCD4 over-expressing stable clones was indicated by the extra band compared with parental cells. pEGFP over-expressing stable clones (433-EV and SKOV3-EV) were also established as the empty vector control and used in the following experiments. The quantification of the western blotting bands was presented in Data S1.

Cell proliferation was assessed by XTT and clonogenic assay. According to the XTT results, both of the two OVCA433-PDCD4 cells exhibited significantly slower proliferation rate compared to the control (p<0.05 for OVCA433-PDCD4c1 and p<0.01 for OVCA433-PDCD4c2, respectively, Figure 1B, left panel). Clonogenic assay also indicated similar results: the numbers of colonies formed for OVCA433-PDCD4c1 and c2 were 33% and 58% less compared to the control (GFP only empty vector control), respectively (p<0.05, Figure 1B, right panel).

To explore the underlying mechanisms for the inhibitory effects of PDCD4 on ovarian cancer cell proliferation, we assessed the effect of PDCD4 on cell cycle progression using flow cytometry analysis. In control OVCA433, the percentage of cells in G1 stage was 69.9% (±3.1%). Comparatively, the percentages of cells in G1 stage were 84.6% (±2.1%) and 80.9% (±2.2%) for OVCA433-PDCD4c1 and c2 respectively, both of which were significantly higher than control (p = 0.004 and P = 0.01, respectively). There was a corresponding decrease of the percentage of cells in the S stage in the OVCA433-PDCD4c1 (10.3%±2.4%) and c2 (15.7%±2%)) compared to control (25.9%±2.1%, Figure 1C).

Over-expression of PDCD4 in SKOV3 also affected its cell cycle progression. In the control SKOV3 cells, the percentage of the cells in G1 stage was 37.5% (±2%). Comparatively, in SKOV3-PDCD4 cells, the percentage of cells in G1 stage was 66.7% (±1.8%), which was significantly higher than control (p = 0.008). There was a corresponding decrease of the percentage of cells in the S stage in SKOV3-PDCD4 cells (20.9%±2.5%) compared to control (46.2%±1.9%).

The flow cytometry analysis indicated that over-expression of PDCD4 induced cell cycle arrest mainly at G1 stage; 1.2 (p< = 0.01) and 1.8 (p<0.01) fold increase in the percentage of the cells at G1 stage in OVCA433-PDCD4 and SKOV3-PDCD4 ovarian cancer cells, respectively (p< = 0.01).

To identify potential molecules involved in the above inhibitory effects of PDCD4 on cell cycle progression, we assessed the expressions of several cell cycle regulators including p21, p27, p53, cyclinA, cyclinE, cdc25a. In OVCA433-PDCD4c1 and c2, p53 expression was barely changed and p21 was significantly up regulated, whereas in p53-null SKOV3-pdcd4 cells, p21 was not detected. P27 expression was up regulated in both OVCA433-PDCD4 and SKOV-PDCD4 cells. In addition, we observed an increase of cdc25a expression in the two 433 PDCD4 over-expressing stable clones, and an increase of cyclinA expression in 433 PDCD4 c2 but not in c1 (Figure 1D). However, only a slightly decrease of cdc25a was observed in SKOV-PDCD4 cell, and cyclinA level was remained the same in both stable clone and vector control of SKOV3 cells. The quantification of the western blotting bands was presented in Data S1.

### PDCD4 inhibited ovarian cancer cell migration and invasion

**Figure 2:**
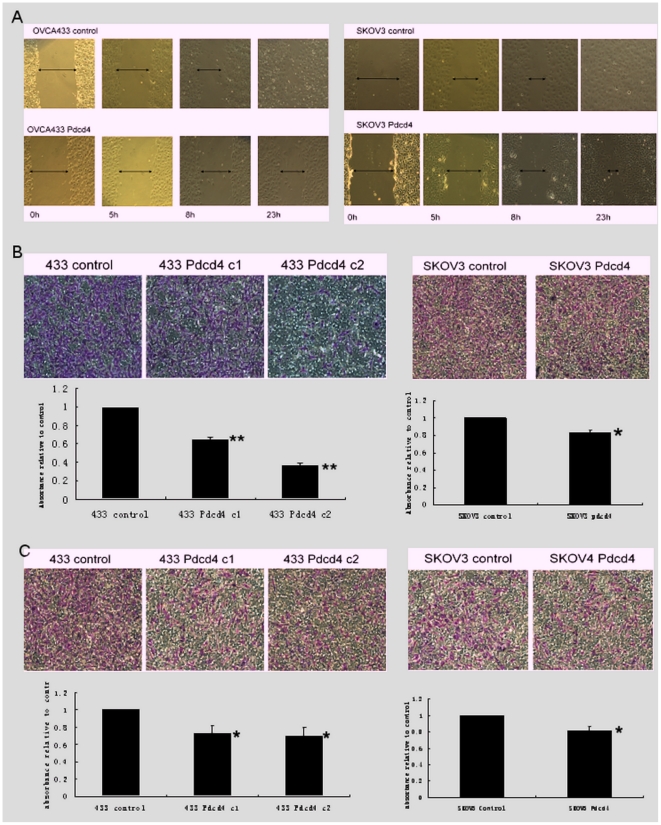
PDCD4 inhibited ovarian cancer cell migration and invasion. (A) Both control and PDCD4 over-expressing cells were treated with mitomycin C (10 ug/ml) for three hours prior to introduction of the wound. Photos were taken at indicated time points (0, 5, 8 and 23 h). PDCD4 over-expressing stable clones exhibited slower wound healing process compared with the control. (B) Ovarian cancer cells were allowed to migrate through the microporous membrane for 9 hours in transwell migration assay. The numbers of cells migrated through for PDCD4 over-expressing stable clones were significantly fewer compared with control (*p<0.01 and **p<0.05, respectively). (C) Ovarian cancer cells were allowed to invade through the ECMatrix for 72 hours in transwell invasion assay. The numbers of cells invaded through for PDCD4 over-expressing stable clones were significantly fewer compared with control (*p<0.05). Experiments were performed in triplicate.

To explore the possible effects of PDCD4 on ovarian cancer cell migration, two different approaches were applied. Firstly, wound healing assay was used to monitor the time required for the closing of the wound in control and PDCD4 over-expressing ovarian cancer cells. Prior to the assay, cells were pre-treated with mitomycin C, a DNA synthesis and nuclear division inhibitor, to ensure the closure of the wound was exclusively due to cell migration but not cell proliferation. The results showed that PDCD4 over-expressing cells exhibited slower migration rate. The scratched wound in both control cells was closed in 23 hours after the introduction of the wound, whereas a gap was still observed in the PDCD4 over-expressing cells (Figure 2A).

To quantitatively assess the cell migration rate, transwell migration assay was applied. OVCA433-PDCD4c1 and c2 showed 35% and 63% less migration, respectively, than that of control cells (p<0.01 Figure 2B). SKOV3-PDCD4 cells showed 22% less migration than that of control cells (p<0.05, Figure 2B).

PDCD4 also has effect on ovarian cancer cell invasion demonstrated by transwell invasion assay. OVCA433-PDCD4c1 and c2 showed 27% and 30% less invasion compared to control (p<0.05 Figure 2C). SKOV3-PDCD4 showed 19% less invasion compared to control (p<0.05, Figure 2C).

### Modulation of PDCD4

**Figure 3:**
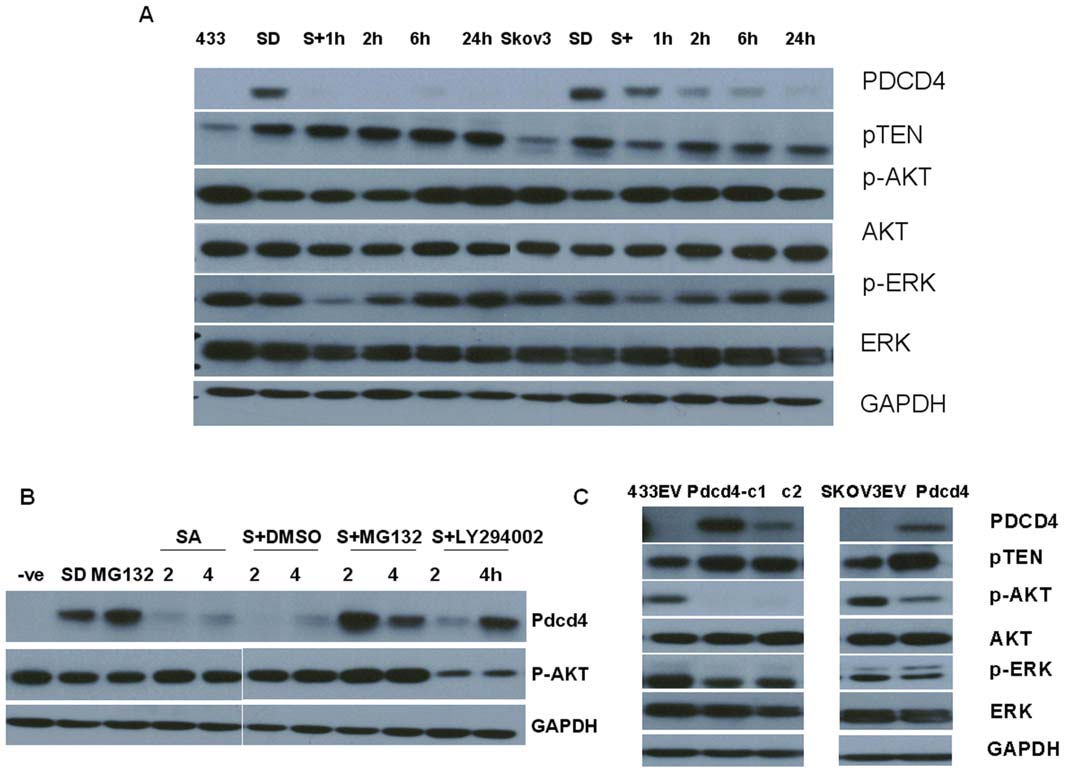
Involvement of p-Akt in the proteasome mediated degradation of PDCD4 during serum deprival-readdition treatment. (A) OVCA433 and SKOV3 were deprived from serum (SD) for 48 hours before serum was re-administrated for 1 h, 2 h, 6 h and 24 h. There was a dramatic increase of PDCD4 protein expression upon serum deprival treatment. PDCD4 rapidly disappeared after serum was re-administered. P-Akt and p-ERK were down-regulated when serum was withdrawn and gradually resumed after serum was added back. Total Akt and ERK were not affected during the treatment. (B) PDCD4 was elevated in serum deprived cells (SD) and depleted when serum was added back (SA, serum addition) for 2 and 4 hours. The administration of either proteasome inhibitor MG132 (S+MG132), or PI3K inhibitor LY294002 (S+LY294002) prevented the depletion of PDCD4. DMSO was also included as control (S+DMSO). Negative control (−ve) indicated for cells cultured with medium containing serum. (C) The expression of PTEN, p-Akt and p-ERK were altered in all PDCD4 over-expression stable clones, whereas the expressions of total Akt and ERK remained unchanged. Three independent experiments were performed. The quantification of the western blotting bands was presented in Data S2.

PDCD4 was reported as a translation inhibitor. As protein translation could be stimulated by serum and inhibited when cells were starved in the absence of growth factors, we thereby examined the effects of serum on the abundance of PDCD4. Both OVCA433 and SKOV3 were starved in serum free medium for 48 hours before serum was re-admitted at indicated time intervals, including 1 h, 2 h, 6 h and 24 h. In both cells, PDCD4 was elevated when starved. However, upon the re-administration of serum, PDCD4 gradually decreased in a time dependent manner in SKOV3 and rapidly disappeared within 1 h in OVCA433 (Figure 3A). The quantification of the western blotting bands was presented in Data S2.

To explore the potential pathways involved in the modulation of PDCD4 in above treatment, the expressions of PTEN, p-Akt and p-ERK (extracellular signal-regulated kinase) were examined. PTEN was elevated after the serum was removed, and there was no further change after re-addition of the serum till up to 24 h. There was a significant decrease of p-Akt when serum was removed in both OVCA433 and SKOV3 cells, followed by resumption at 6 h after serum re-addition in OVCA433. In SKOV3, the recovery of p-Akt was as fast as 1 h. A slight decrease of p-ERK was observed in both cells in the absence of serum, and a further reduction was also observed in both cells when serum was re-admitted for 1 h. The expression of p-ERK was resumed after 2 h of serum administration and gradually reached to the original level at 24 h. No profound change was observed in either total Akt or ERK (Figure 3A).

To confirm the potential involvement of PI3K-Akt and MEK-ERK pathways in the regulation of PDCD4, Specific PI3K inhibitor LY294002, and MEK inhibitor U0126 were introduced to the starved cells together with serum and incubated for 2 h and 4 h after 48 hours' starvation treatment. When p-Akt was specifically down regulated upon the treatment of LY294002, the depletion of PDCD4 was prevented (Figure 3B). However, the administration of U0126 did not exhibit any effect on the prevention of PDCD4 degradation (Figure S1). In addition, when proteasome inhibitor MG132 was introduced to the starved cells, the depletion of PDCD4 was also prevented (Figure 3B). No effect was observed in DMSO control cells. Our results indicated that the depletion of PDCD4 after the re-addition of serum in ovarian cancer cells was due to the proteasome-mediated degradation. The quantification of the western blotting bands was presented in Data S2.

In the PDCD4 over-expressing ovarian cells, PTEN was up regulated, and p-Akt and p-ERK was significantly down regulated, whereas total Akt and ERK were not affected (Figure 3C). The quantification of the western blotting bands was presented in Data S2.

### Intracellular translocation of PDCD4

**Figure 4:**
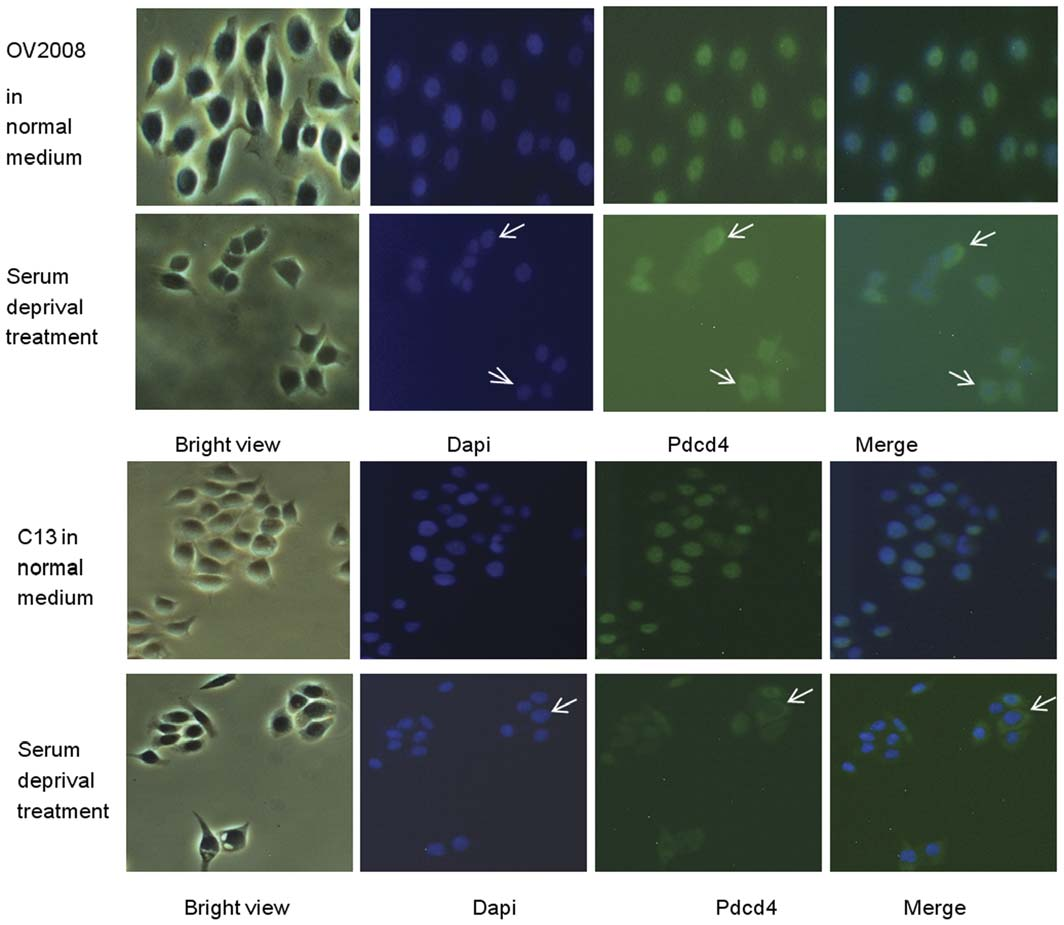
Intracellular translocation of PDCD4 under normal and serum-free culture medium. Endogenous PDCD4 in both OV2008 and C13 ovarian cancer cells was localized exclusively in the nucleus when cultured in medium with serum. More cytoplasmic localization of PDCD4 was observed under serum starvation treatment. Endogenous PDCD4 was detected by immunofluorescent staining using PDCD4 primary antibody and FITC-labeled goat anti-rabbit secondary antibodies. Dapi staining indicated nuclear localization. Representative translocation was indicated by arrows.

Our previous immunohistochemistry study showed a differential cellular localization pattern of PDCD4 between normal and malignant ovarian cells [17], suggesting that PDCD4 might translocate from the nucleus to the cytoplasm during ovarian cancer development. Other study also demonstrated the intracellular translocation of PDCD4 under some stress conditions [18]. We then investigated the endogenous PDCD4 localization in ovarian cancer cells. Two ovarian cancer cell lines, OV2008 and C13, have high expression level of the endogenous PDCD4, which was found localized exclusively in the nucleus under normal culture condition (Figure 4). After serum starvation treatment for 48 hours, the endogenous PDCD4 was found to be translocated from nucleus to the cytoplasm (Figure 4).

## Discussion

A number of studies have reported the inhibitory effects of PDCD4 on protein translation [19], [20], and AP-1 dependent transactivation [12], [21]. Studies on PDCD4's function on cell cycle have generated inconsistent results. In breast cancer cells, PDCD4 caused an increased population in G0 to G1 phase without affecting other phases of cell cycle suggesting a potential role in apoptosis [22]. In Glioma cancer cells, PDCD4 delayed cell cycle transition from G1-to-S phase [23]. Another group reported that PDCD4 induced cells in both sub-G1 and G2-S phase, indicating its effects on both apoptosis and cell cycle arrest [24]. However, in colon cancer cells, PDCD4 did not alter cell cycle progression or induce apoptosis [8]. In the current study, ectopic PDCD4 expression induced cell cycle arrest at G1 stage and consequently suppressed ovarian cancer cell proliferation.

We assessed the expression of a panel of cell cycle regulators that play important roles in G1-S transition in PDCD4 over-expressing cells. PDCD4 induced the expression of p27 and p21, but not the expressions of cyclin E. Our results were consistent with the findings that knockdown of PDCD4 in AML cells resulted in down regulation of p27 [25]; and the induction of p21 and p27 by PDCD4 [26]. The cellular effects of PDCD4 were suggested to be different in cell models with or without p53 expression [10], [27]. Two ovarian cell lines selected in this study have differential p53 status, OVCA433 bearing wild type p53, and SKOV3 being null p53. Both cell lines had elevated p27 expressions upon overexpression of PDCD4, and the up-regulation of p21 was not mediated by p53 in OVCA433 cells. Similar findings have been reported that induction of p21 by PDCD4 in carcinoid cells was independent of p53 [7]. Our findings implied that one of the potential mechanisms for the induction of cell cycle arrest at G1 stage by PDCD4 was due to the up regulation of p21 and p27. We noticed that there was no difference in cyclin A or cdc25a expressions between stable clone and empty vector control in SKOV3 cell lines. However, for OVCA433 cells, we observed an increase of cdc25a expression in the two PDCD4 over-expressing stable clones, and an increase of cyclin A expression in 433 PDCD4 c2 but not in c1. The differential expression of cyclin A may be due to the different proliferative and clonogenic activities of PDCD4-expressing clone c1 and c2. Nevertheless, the effects of PDCD4 on cyclin A and cdc25a would need further investigation.

Besides proliferation, we also demonstrated the inhibitory effects of PDCD4 on ovarian cancer cell migration and invasion. Similar effects have also been reported in studies conducted in colon and HCC cells [8], [20], [28]. PDCD4 was found to be induced upon the treatment of pro-apoptotic substances [29], [30]. Investigations on its role in apoptosis have generated inconsistent results. PDCD4 has been demonstrated to induce apoptosis in breast and lung cancer cells [22], [26] In contrast, no apoptotic effect of PDCD4 was observed in other studies [8], [12]. Additionally, higher PDCD4 expressions were reported to be correlated with increased sensitivity to geldanamycin and tamoxifen [31]. Moreover, a recent study conducted in prostate cancer cells suggested an increased cisplatin and paclitacel sensitivity by over-expression of PDCD4 [32]. In the present study, overexpression of PDCD4 did not induce apoptosis according to flow cytometry and western blot assay through PARP expression (Figure S2). We also assessed the potential role of PDCD4 on cisplatin sensitivity. However, no significant difference was observed between PDCD4 over-expressing cells and the control cells in response to cisplatin treatment (Figure S2), which was consistent to our previous published data that no correlation of PDCD4 expression with chemosensitivity status of ovarian cancer patients was observed [17]. Given that the mechanism for cisplatin to kill cancer cells is to initiate cell apoptosis, and PDCD4 showed no apoptotic effect in ovarian cancer cells, this might be one of the reasons contributing to the above-mentioned observation.

As noted from our results, the magnitude of suppression of ovarian cancer cell proliferation, migration and invasion was not in proportion to the over-expression levels of PDCD4 protein in those stable clones. Similar findings were also reported in the study conducted by Yang et al. in mouse epidermal JB6 RT101 cells [21]. We proposed that there might be a concentration threshold, when exceeded, the inhibitory function of PDCD4 on tumor progression would occur. According to our flow cytometry assessment, over-expression of PDCD4 in both OVCA433 and SKOV3 cells induced cell cycle arrest at G1 stage. However, although a suppressive effect on cell proliferation and colony formation was observed in PDCD4 over-expressing SKOV3 cells, the effect was not statistical significant when comparing to the parental control cells (Figure S3). Whether it was due to the genetic background of SKOV3 cells or the involvement of other mechanisms and factors were currently not clear and required further investigation.

The observations of the down-regulation of p-Akt and p-ERK in PDCD4 over-expressing cells suggested a potential involvement of p-Akt and p-ERK pathways in the regulation of PDCD4. To address the question that whether these two pathways are actually involved, we proceeded to study the concurrent expressions of p-Akt, p-ERK and PDCD4 during the serum withdrawal and re-addition treatment. There was a dramatic increase of PDCD4 upon serum withdrawal, followed by rapid depletion of PDCD4 after serum was re-administered. During the same process, a reverse trend of the expression of both of p-Akt and p-ERK, the two important cell proliferation regulatory molecules, was observed: an initial decreased expression followed by a gradual resumption. The alteration of p-Akt and p-ERK might be simply the consequences of serum deprival treatment. And still, given the concurrent changes of the expressions of PDCD4 and p-Akt and p-ERK, and the altered expression level of both molecules observed in the PDCD4 over-expressing cells, there is a possibility that p-Akt and p-ERK pathways was involved in the regulation of PDCD4. To further confirm it, we applied PI3K inhibitor LY294002 (which subsequently blocks Akt phosphorylation) together with serum, and found PDCD4 protein was no longer degraded. Introduction of MEK inhibitor did not prevent the depletion of PDCD4. Our results indicated that p-Akt but not p-ERK was required for the degradation of PDCD4 upon serum stimulation, and thus PI3K-Akt pathway might play a direct role on the modulation of PDCD4 degradation in ovarian cancer cells. However, the involvement of p-ERK pathway is not clear at the present and required further investigation. There have been studies reporting the p-Akt pathway on the regulation of PDCD4. Dorrello's study implicated the role of S6K1 in the regulation of PDCD4 degradation in response to mitogen [23]. Treatment with tumor promoter 12-O-tetradecanoylphorbol-13-acetate (TPA) decreased PDCD4 expression, which was attributable to proteasomal degradation mediated by PI3K-Akt-mTOR-p70^S6K^ and facilitated by MEK-ERK signaling pathway [16]. An inverse correlation of PDCD4 and p-Akt expression has been reported in colorectal cancer tissue samples [33]. shRNA PDCD4 activated Akt pathway, leading to the increased expressions of p-Akt, mTOR and p70S6K in the lungs of mice [34]. Our results were in concordance to these findings and implied the involvement of p-Akt pathway in the regulation of PDCD4 in ovarian cancer cells.

**Figure 5:**
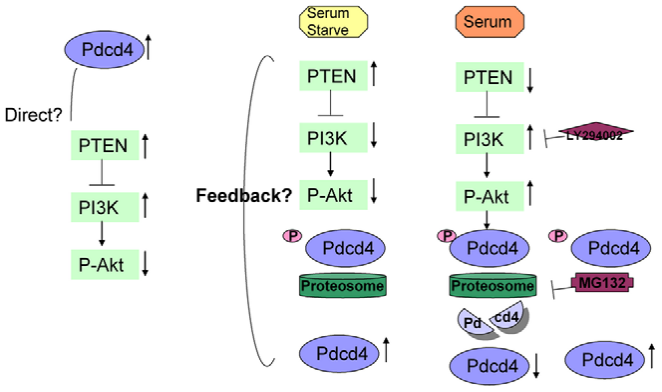
Involvement of p-Akt pathway in the modulation of PDCD4. Elevated PTEN and suppressed p-Akt were found in the PDCD4 over-expressing stable clones. When cells were starved with serum-free medium, PDCD4 was un-phosphorylated due to suppressed p-Akt expression. Un-phosphorylated PDCD4 was not recognized by proteasome thus leading to the accumulation of PDCD4. When serum was re-administrated to the cells, up-regulated p-Akt phosphorylated PDCD4, which was then depleted through proteasome degradation. The administration of either PI3K inhibitor LY294002, capable of preventing PDCD4 from being phosphorylated by p-Akt, or proteasome inhibitor MG132, preventing the depletion of PDCD4, leading to the accumulation of PDCD4.

Combining the phenomenon that down-regulation of p-Akt expression was observed in PDCD4 over-expressing cells, and the fact that blocking of p-Akt by PI3K inhibitor prevented the degradation of PDCD4 during the serum starvation and re-addition process, we proposed a potential feedback control of PDCD4 through p-Akt pathway: establishment of PDCD4 over-expressing stable clones could only be achieved when degradation of PDCD4 was inhibited, which required suppression of p-Akt expression (Figure 5). However, the potential mechanisms mediating this feedback control and molecules involved have not been identified and further investigations are required.

Our current results demonstrated a translocation of endogenous PDCD4 from nucleus to cytoplasm upon serum starvation. We hypothesized that PDCD4 might shuttle to the cytoplasm to exhibit its inhibitory function in protein translation when cells were under unfavorable growth condition. According to our previous findings, a differential localization of PDCD4 was observed between normal and malignant ovarian tissue samples: more cytoplasmic localization of PDCD4 was observed in ovarian cancer tissue samples [17]. When tumour grows, there may be some regions where the oxygen concentration is significantly lower than that in the normal tissues, and tumour cells are under a hypoxic stress. One hypothesis is that in these cells, PDCD4 may shuttle to the cytoplasm to inhibit translation and subsequently cell growth. On the whole, PDCD4 functions as tumor suppressor in the nucleus in normal cells, however, when cells were under certain environmental stress or undergoing potential transformation from normal to malignant, one of the cellular response might be transporting PDCD4 to the cytoplasm. This renders the localization of PDCD4 as an indicator of cells under abnormal growth environment or neoplasmic transformation. Nevertheless, the translocation mechanism of PDCD4 is still not yet clear and the hypothesis needs to be further evaluated.

## Materials and Methods

### Cell culture, serum starvation treatment and drug treatment

Ovarian cancer cell lines OVCA433 and SKOV3 used in this study were gift from Prof. SW Tsao, Department of Anatomy, the University of Hong Kong. Ovarian cancer cell lines C13 and OV2008 were gift from Prof. BK Tsang, Department of Obstetrics and Gynaecology, University of Ottawa, Canada. These cell lines were cultured in MEM, with 10% Fetal Bovine Serum (Invitrogen Corporation, Carlsbad, CA).

Cells under serum starvation treatment were cultured in MEM without FBS. Phosphoinositide 3-kinase (PI3K) inhibitor LY294002 (Cell Signaling Technology Inc., Danvers, MA), MEK inhibitor U0126 (Cell Signaling), proteasome inhibitor MG132 (Cell signaling) were dissolved in DMSO (Sigma Co., St. Louis, MO) and further diluted before introduced to cells. Medium with DMSO alone was also included as the control.

### Antibodies and western blot analysis

Protein extraction and western blot methods were the same as previously described [17]. PDCD4 antibody was from Rockland (Rockland immunochemicals, Gilbertsville, PA); antibodies of p21, cyclinA, cyclinE and p53 were from Santa Cruz; antibodies of PTEN, p-Akt, Akt, p-ERK, ERK, p-JNK, JNK, cdc25a, mTOR were from cell signaling; antibody of p27 was from BD Transduction laboratories.

### Construction of PDCD4 cDNA expression vector

PDCD4 full-length cDNA was amplified by PCR using Human PDCD4 cDNA clone (OriGene Technologies, Inc., Rockville, MD) (genebank accession number NM_014456.3) as the template and the following primer pairs 5′ gaattccATGGATGTAGAAAATGAGCAGA 3′ (sense) and 5′ gtcgacTCAGTAGCTCTCTGGTTTAAGA 3′ (antisense), which contained ECORI and SalI restriction enzyme sites. A 1.5-kb, full-length PDCD4 PCR product was then cloned in frame, into pEGFP-C1 (Clontech laboratories, Mountain View, CA).

### Establishment of PDCD4 over- expressing stable clones

PDCD4 plasmid or GFP-C1 vector was transfected into OVCA433 and SKOV3 ovarian cancer cells using FUGENE HD transfection reagent (Roche molecular biochemicals, Manniheim, Germany) according to the manufacturer's protocol. Cells were then harvested for western blot analysis or assay. For PDCD4 and GFP empty vector stable clone development, cells were further treated with geneticin (G418) (Gibco) for two weeks until single colonies could be picked and established stable clones were further assessed and confirmed by western blot.

### Cell proliferation assay

Cell proliferation was assessed by both XTT and clonogenic assay. XTT assay (Roche) was performed according to the manufacturer's protocol. Briefly, 1000 cells were seeded in 96-well plate and treatment was initiated on the next day. Cells were further cultured and harvested on four consecutive days. Harvested cells were incubated with XTT labeling mixture for 4 hours. The absorbance was quantitated by fluorescence microplate reader (Infinite F200, Tecan Group Ltd., Männedorf, Switzerland).

Clonogenic assay was used to test cell viability over a relatively longer period of time. Cells with or without cisplatin treatments were seeded at a concentration of 450 cells per well in 6-well plates and allowed to grow for 9 days. Cells were fixed with 70% ethanol and stained with 1% Giemsa (Merck). Colonies consisting of more than 50 cells were counted.

### Cell cycle analysis by flow cytometry

Cells were harvested, washed and fixed in 70% ethanol at 4°C overnight, followed by incubation with RNaseA (100 ug/ml) (Roche) to remove RNA contamination. Propidium iodide (Calbiochem, Merck Biosciences) was then added in and the DNA contents and cell cycle distributions were analyzed using an EPICS Elite ESP flow cytometer (Beckman Coulter Ltd.). The cell cycle was analyzed by Winlist and cylchred software.

### Wound healing assay, cell migration and invasion assay

Wound healing assay was used to assess the cell migration in vitro. Cells were seeded and allowed to grow until 100% confluence was reached. Mytomycin C (Sigma) was introduced 3 hours prior to the start of the assay to inhibit cell proliferation. A wound was then generated by scratching a straight line. Cells were washed to remove dislodged cells and mitomycin C-containing medium was replaced with normal medium. Migration of cells into denuded areas was monitored and visualized by a phase contrast microscope.

To quantitatively assess cell migration rate, QCM 24-well colorimetric cell migration assay kit (Millipore, Billerica, MA) was applied according to the manufacturer's protocol. Briefly, cells were starved for 18 hours prior to the assay in serum-free MEM. 150,000 cells were added to the inner chamber and MEM with 10% FBS was added to the outside chamber. Cells were further incubated for 9 hours. Cells migrated through were stained, extracted and then measured at the optical density at 560 nm. The CHEMICON Cell Invasion Assay Kit (Millipore) was used to quantitatively assess cell invasion. Similar procedures were applied and cells were allowed to invade through the chamber for 72 hours before harvested for analysis.

### Immunofluorescent staining

Localization of endogenous PDCD4 was assessed by immunofluorescent staining. Ovarian cancer cells OV2008 and C13 were seeded on the sterile cover slip staged in a 6-well plate. After attachment and treatment, cells were fixed in 4% paraformaldehyde (USB Corporation, cleverland, OH) in PBS (pH 7.4), permeabilised in permeabilization solution (0.1% Triton X-100 in 0.1% sodium citrate, Sigma) and then blocked with 3% BSA in PBS, followed by incubation with PDCD4 primary antibody (1∶500 dilution with PBS containing 3% BSA). FITC conjugated AffiniPure Goat anti-rabbit IgG secondary antibody (Jackson ImmunoResearch Laboratories Inc., West Grove, PA) was then applied. Slides were then stained with Dapi and coverslips were mounted with fluorescent medium VEITASHIELD (Vector Laboratories Inc., Burlingame, CA) to preserve the fluorescent signal. The localization of endogenous PDCD4 was visualized by fluorescent microscope.

### Statistical analysis

Three independent experiments were performed for each analysis (including XTT, clonogenic assay, flow cytometry analysis, migration assay, invasion assay and western blot). Data collected from cell proliferation (XTT and clonogenic assays), apoptosis and flow cytometry analysis was processed by Microsoft Excel. Data was transformed into percentage control and presented as mean±SEM (for flow cytometry analysis) or in either bar (for migration and invasion assay) or line charts (for XTT proliferation assay). Graph for clonogenic assay data was constructed according to the actual colony numbers formed by the end of the assay. Differences between groups were assessed by two-tailed student t-test and were considered to be significant at p<0.05.

## Supporting Information

Figure S1 **MEK inhibitor U0126 did not prevent PDCD4 degradation upon serum readdition treatment.** PDCD4 was elevated in serum deprived cells (SD) and depleted when serum was added back (SA, serum addition) for 2 and 4 hours. The administration MEK inhibitor U0126 (S+U0126) did not prevent the depletion of PDCD4.
(TIF)

Figure S2 **PDCD4 did not induce apoptosis or chemoresponse in ovarian cancer cells.** PARP expression was assessed by western blot in PDCD4 over-expressing stable clones in ovarian cancer cells SKOV3 (SKOV3 PDCD4) and OVCA433 (c1 and c2) as well as control cells without (A) or with (B) cisplatin treatment (15 uM, 48 h). Positive controls (+ve) were cells with cisplatin treatment (15 uM, 48 h). Apoptosis was indicated by the additional band (cleaved PARP) in addition to the full length PARP. No difference on cleaved PARP was observed between PDCD4 over-expressing stable clones and control cells either with or without cisplatin treatment.
(TIF)

Figure S3 **The effect of PDCD4 on cell proliferation of SKOV3 ovarian cancer cells.** A suppressive effect on cell proliferation indicated by XTT assay (A) and colony formation assay (B) was observed in PDCD4 over-expressing SKOV3 cells. However, the effect was not statistical significant when comparing to the parental control cells.
(TIF)

Data S1 **Three independent experiments were performed for all the western blot studies.** The intensity of the western blot band was determined by densitometric scanning. The quantitative analysis of the western blot data for Figure 1A and Figure 1C was presented in Data S1. Y-axis indicated the relative band densities of the target proteins in PDCD4 over-expressing stable clones compared with control (PDCD4 parental cells or cells transfected with empty vector).
(DOC)

Data S2 **Three independent experiments were performed for all the western blot studies.** The intensity of the western blot band was determined by densitometric scanning. The quantitative analysis of the western blot data for Figure 3A, Figure 3B and Figure 3C was presented in Data S2. Y-axis indicated the relative band densities of the target proteins in PDCD4 over-expressing stable clones compared with control (PDCD4 parental cells or cells transfected with empty vector).
(DOC)
