# Supplementary figures and images for: Tumour Suppressive Function and Modulation of Programmed Cell Death 4 (PDCD4) in Ovarian Cancer
Source: PLoS One. 2012 Jan 17;7(1):e30311. doi: 10.1371/journal.pone.0030311 (PMC3260274; doi:10.1371/journal.pone.0030311)

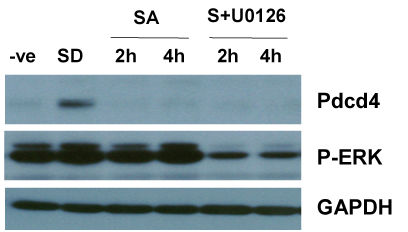

Supplement: Figure S1 — MEK inhibitor U0126 did not prevent PDCD4 degradation upon serum readdition treatment. PDCD4 was elevated in serum deprived cells (SD) and depleted when serum was added back (SA, serum addition) for 2 and 4 hours. The administration MEK inhibitor U0126 (S+U0126) did not prevent the depletion of PDCD4. (TIF) [file pone.0030311.s001.tif]

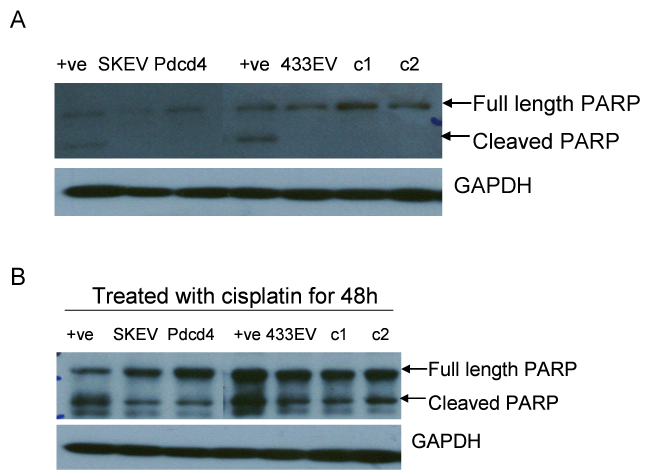

Supplement: Figure S2 — PDCD4 did not induce apoptosis or chemoresponse in ovarian cancer cells. PARP expression was assessed by western blot in PDCD4 over-expressing stable clones in ovarian cancer cells SKOV3 (SKOV3 PDCD4) and OVCA433 (c1 and c2) as well as control cells without (A) or with (B) cisplatin treatment (15 uM, 48 h). Positive controls (+ve) were cells with cisplatin treatment (15 uM, 48 h). Apoptosis was indicated by the additional band (cleaved PARP) in addition to the full length PARP. No difference on cleaved PARP was observed between PDCD4 over-expressing stable clones and control cells either with or without cisplatin treatment. (TIF) [file pone.0030311.s002.tif]

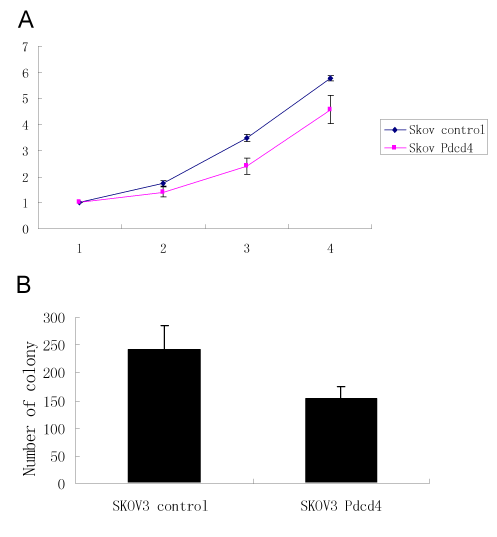

Supplement: Figure S3 — The effect of PDCD4 on cell proliferation of SKOV3 ovarian cancer cells. A suppressive effect on cell proliferation indicated by XTT assay (A) and colony formation assay (B) was observed in PDCD4 over-expressing SKOV3 cells. However, the effect was not statistical significant when comparing to the parental control cells. (TIF) [file pone.0030311.s003.tif]
