# Supplementary material for: Tumour Suppressive Function and Modulation of Programmed Cell Death 4 (PDCD4) in Ovarian Cancer
Source: PLoS One. 2012 Jan 17;7(1):e30311. doi: 10.1371/journal.pone.0030311 (PMC3260274; doi:10.1371/journal.pone.0030311)

Data S1

Three independent experiments were performed for all the western blot studies. The intensity of the western blot band was determined by densitometric scanning. The quantification of the bands was presented below. Y-axis indicated the relative band densities of the target proteins in PDCD4 over-expressing stable clones compared with control (PDCD4 parental cells or cells transfected with empty vector).

Quantitative analysis of the western blot data for Figure 1A


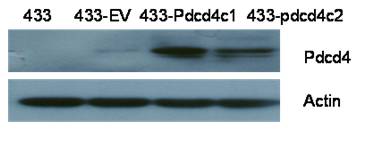

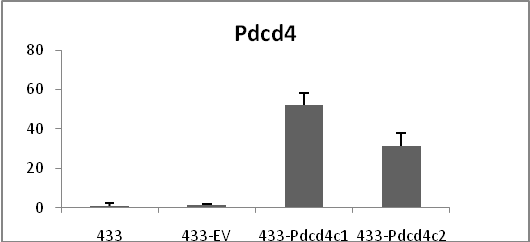


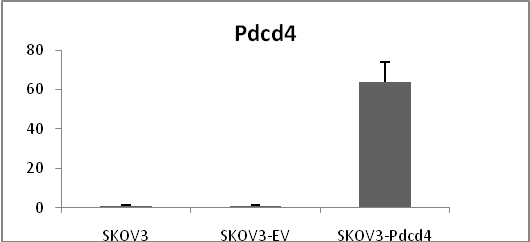

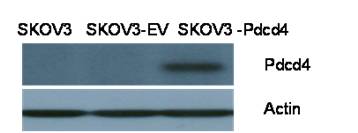


Quantitative analysis of the western blot data for Figure 1D


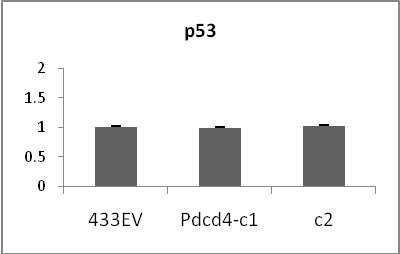
433EV Pdcd4-c1 c2


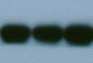


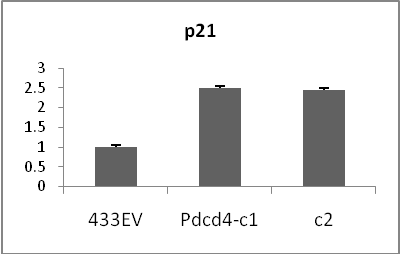


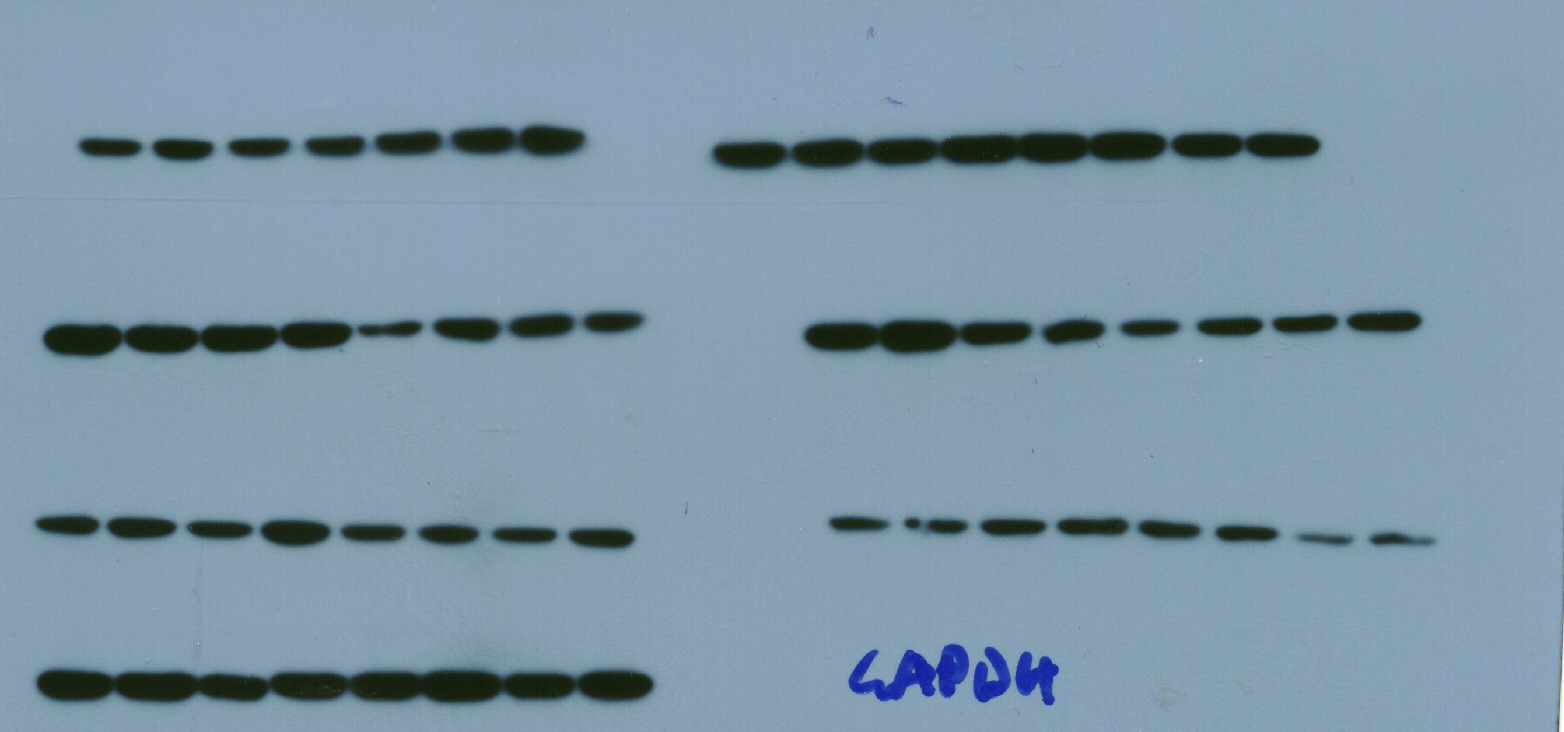


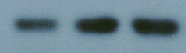

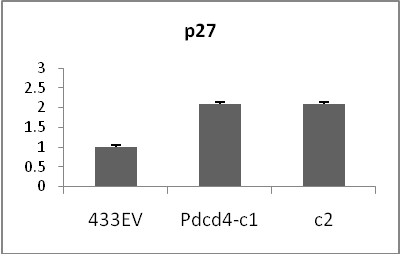


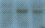

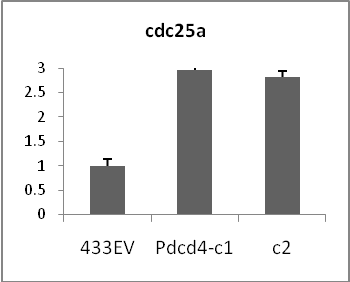


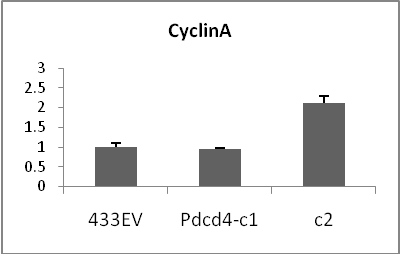

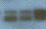


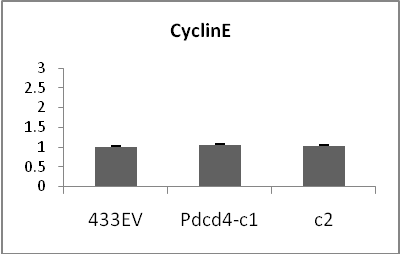


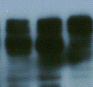


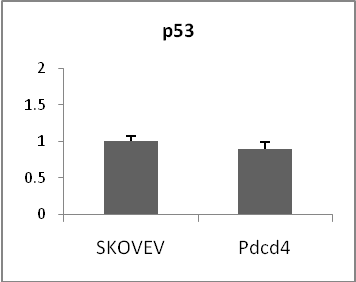
SKOVEV Pdcd4


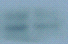


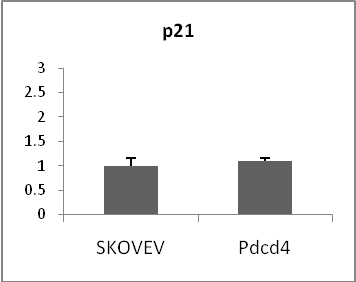

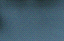


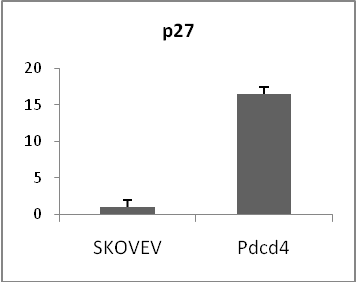


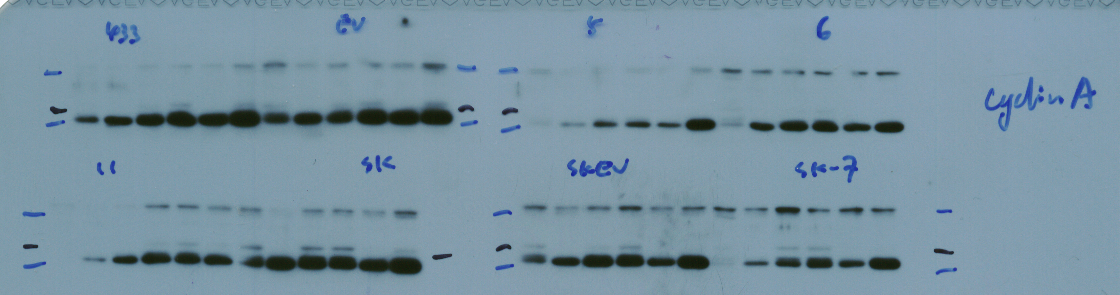


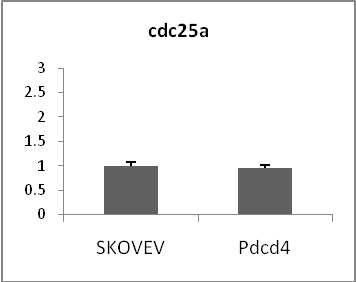


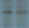


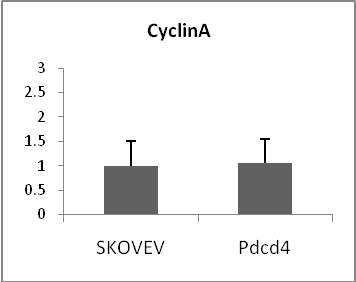


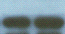


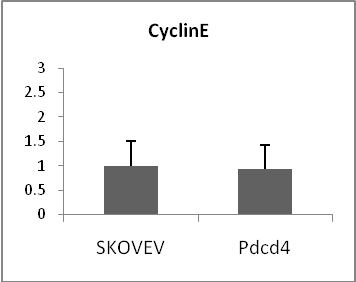


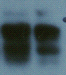

Supplement: Data S1 — Three independent experiments were performed for all the western blot studies. The intensity of the western blot band was determined by densitometric scanning. The quantitative analysis of the western blot data for Figure 1A and Figure 1C was presented in Data S1. Y-axis indicated the relative band densities of the target proteins in PDCD4 over-expressing stable clones compared with control (PDCD4 parental cells or cells transfected with empty vector). (DOC) [file pone.0030311.s004.doc]
