# Supplementary material for: Tumour Suppressive Function and Modulation of Programmed Cell Death 4 (PDCD4) in Ovarian Cancer
Source: PLoS One. 2012 Jan 17;7(1):e30311. doi: 10.1371/journal.pone.0030311 (PMC3260274; doi:10.1371/journal.pone.0030311)

Data S2

Three independent experiments were performed for all the western blot studies. The intensity of the western blot band was determined by densitometric scanning. The quantification of the bands was presented below. Y-axis indicated the relative band densities of the target proteins in PDCD4 over-expressing stable clones compared with control (PDCD4 parental cells or cells transfected with empty vector).

Quantitative analysis of the western blot data for Figure 3A


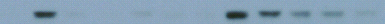

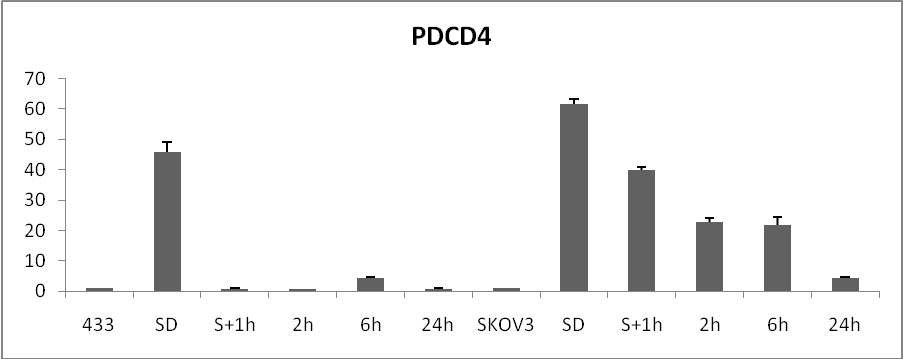


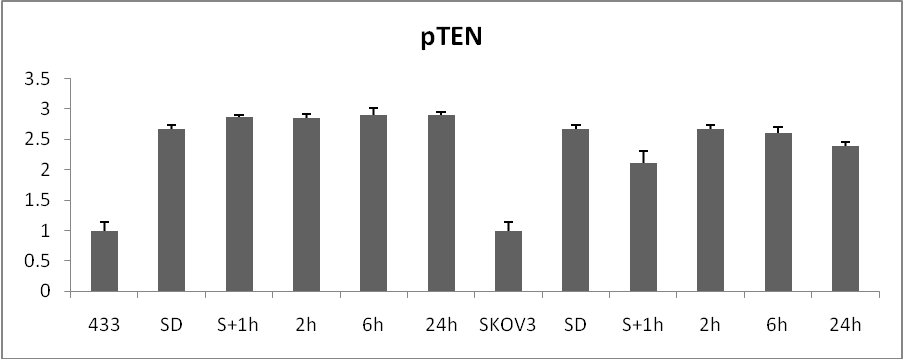


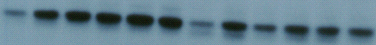


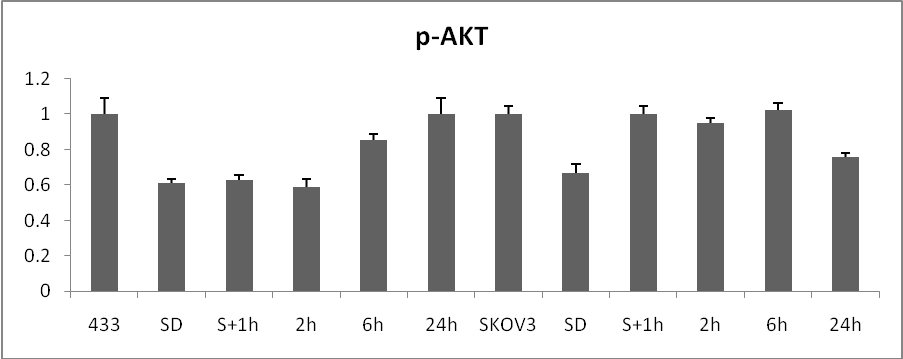


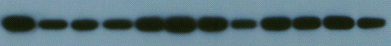


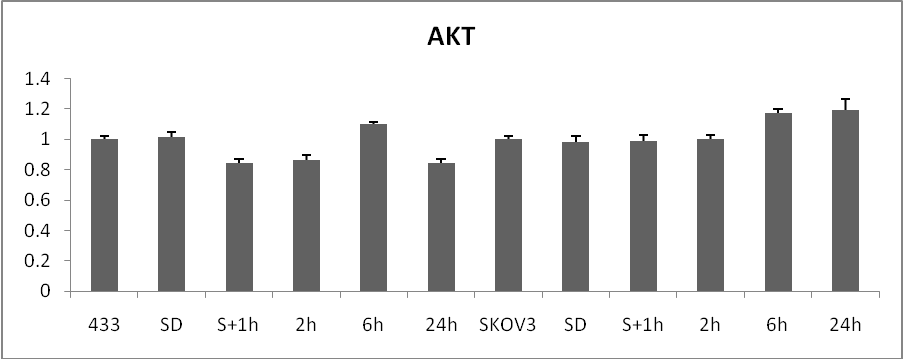

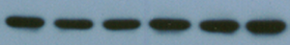

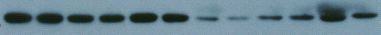


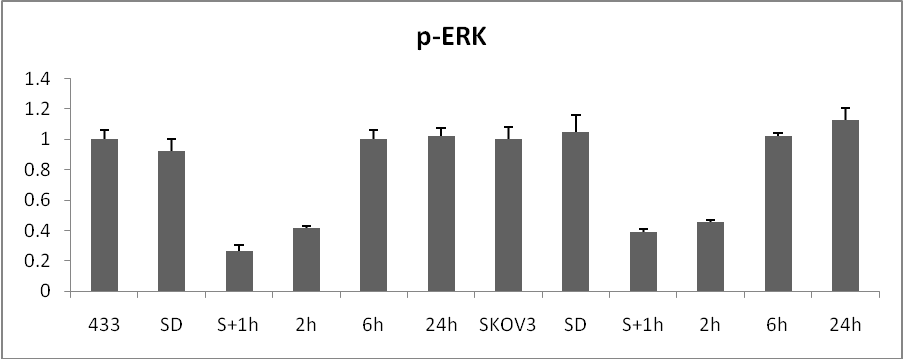


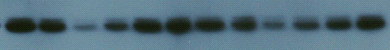


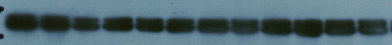

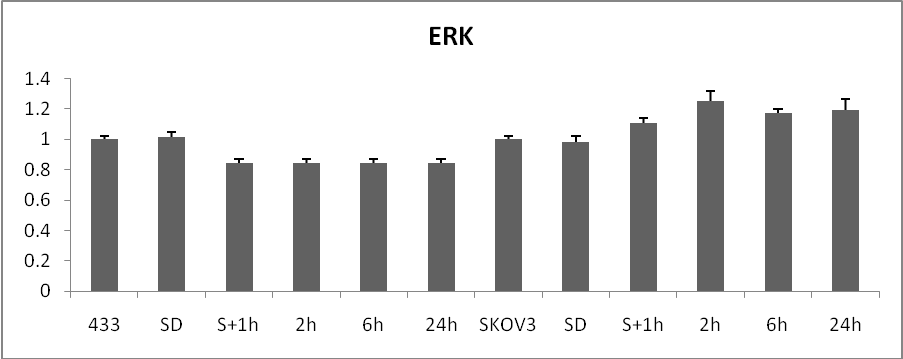


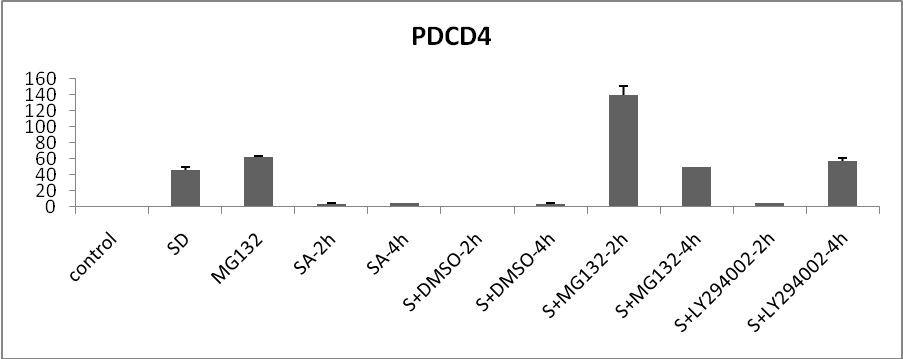
Quantitative analysis of the western blot data for Figure 3B


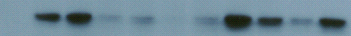


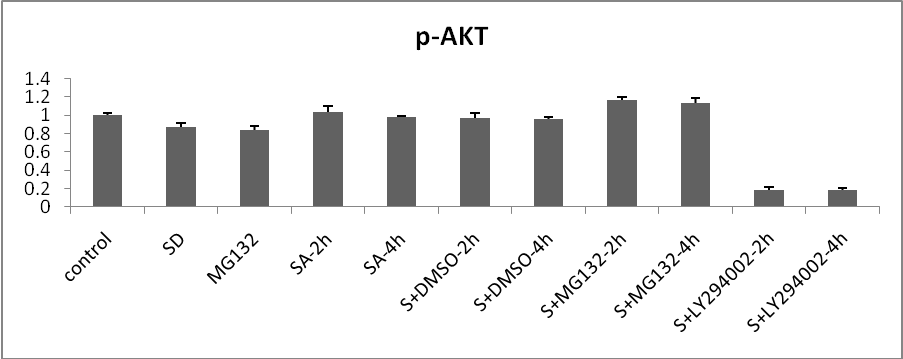


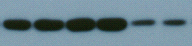

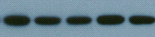


Quantitative analysis of the western blot data for Figure 3C

433EV Pdcd4-c1 c2


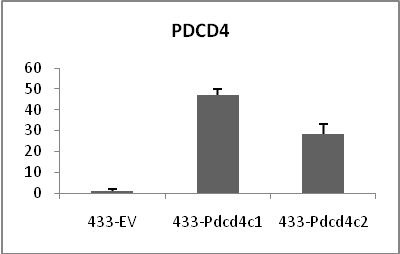

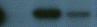


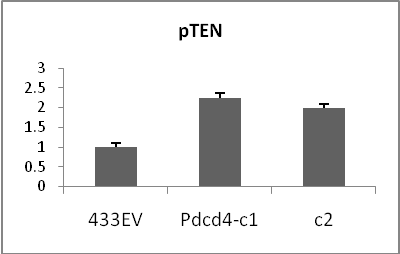

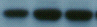


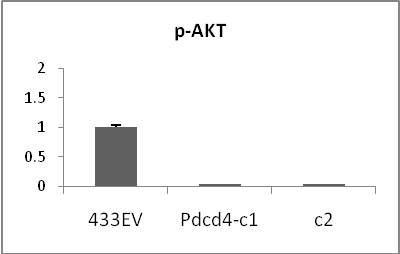

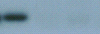


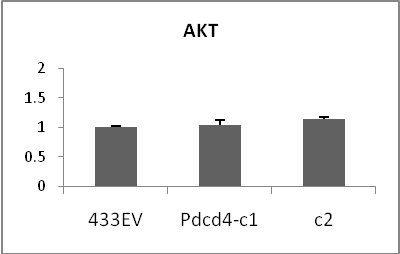

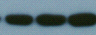


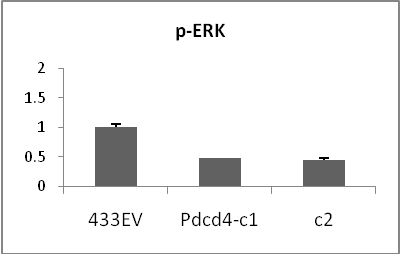

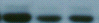


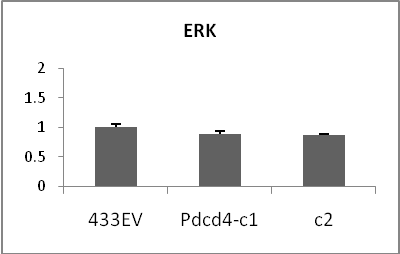


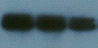


SKOVEV Pdcd4


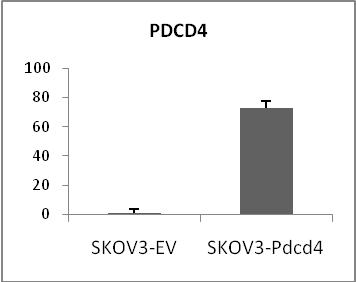

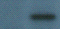


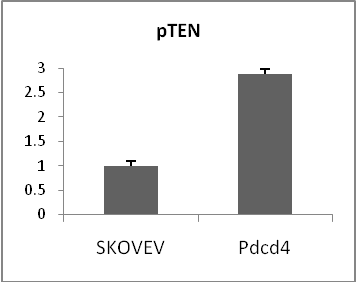

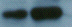


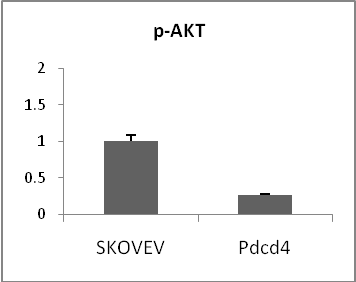


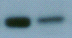


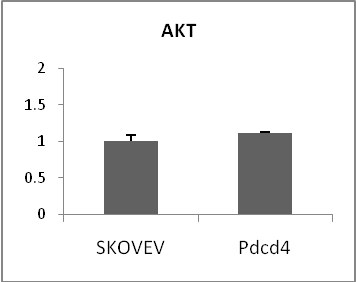


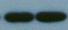


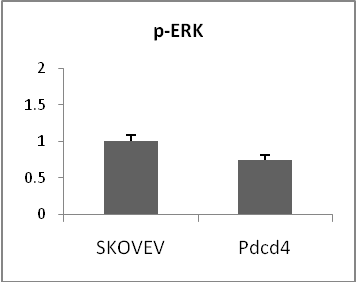


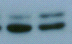


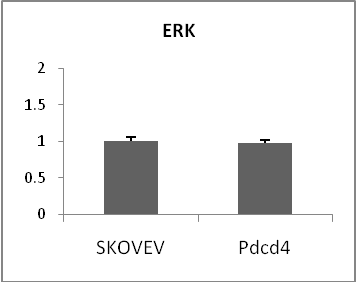

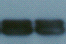

Supplement: Data S2 — Three independent experiments were performed for all the western blot studies. The intensity of the western blot band was determined by densitometric scanning. The quantitative analysis of the western blot data for Figure 3A, Figure 3B and Figure 3C was presented in Data S2. Y-axis indicated the relative band densities of the target proteins in PDCD4 over-expressing stable clones compared with control (PDCD4 parental cells or cells transfected with empty vector). (DOC) [file pone.0030311.s005.doc]
